# Unsupervised classification for region of interest in X-ray ptychography

**DOI:** 10.1038/s41598-023-45336-4

**Published:** 2023-11-13

**Authors:** Dergan Lin, Yi Jiang, Junjing Deng, Zichao Wendy Di

**Affiliations:** 1 Mathematics and Computer Science Division, Argonne National Laboratory, Lemont, IL 60439 USA; 2 Advanced Photon Source, Argonne National Laboratory, Lemont, IL 60439 USA

**Keywords:** Mathematics and computing, Optics and photonics

## Abstract

X-ray ptychography offers high-resolution imaging of large areas at a high computational cost due to the large volume of data provided. To address the cost issue, we propose a physics-informed unsupervised classification algorithm that is performed prior to reconstruction and removes data outside the region of interest (RoI) based on the multimodal features present in the diffraction patterns. The preprocessing time for the proposed method is inconsequential in contrast to the resource-intensive reconstruction process, leading to an impressive reduction in the data workload to a mere 20% of the initial dataset. This capability consequently reduces computational time dramatically while preserving reconstruction quality. Through further segmentation of the diffraction patterns, our proposed approach can also detect features that are smaller than beam size and correctly classify them as within the RoI.

## Introduction

X-ray transmission ptychography is a coherent diffractive imaging (CDI). It can achieve nanoscale resolution through measuring multiple diffraction patterns by scanning an X-ray probe across an object of interest, where the resolution is limited only by the spatial frequency of diffracted signals^1–3^. Unlike conventional CDI that takes a diffraction pattern from an isolated sample^4,5^, ptychography can image an extended sample by scanning the sample through a finite illumination to acquire a series of diffraction patterns. Typical ptychography experiments collect a large number of diffraction patterns to cover an extended region with sufficient overlapping to achieve high-resolution reconstruction images. However, traditional 2D scans used to capture the object typically include a large portion of unwanted or unnecessary regions, for example, empty background, thus imposing a high expenditure of computational resources on the reconstruction of regions that may not be of interest. Currently in X-ray ptychography, in order to reduce the data acquired from an area that is not a region of interest (RoI), an initial coarse scan on a large field of view (FoV) is performed to estimate the object’s RoI position. This is then followed by a finer scan on a smaller FoV. While this approach reduces the scanning region, a rectangular or square scan FoV can still cover a significant portion of unwanted data points, especially when the scanned object is a particle or cell type. In some cases, such as ptychographic tomography, additional empty regions are even added on purpose to increase the scan FoV to accommodate sample drifts or rotation errors, which are expected to be uninformative for the reconstruction. Therefore, it is highly desirable to extract only the informative data points needed to perform a satisfying ptychographic reconstruction with minimum computational cost.

Various techniques have been developed that use information directly extracted from diffraction patterns to approximate the desired information regarding an object. For example, in the aforementioned approach of pre-coarse scan to estimate the RoI for ptychography, the absorption contrast can be obtained from the total transmitted intensity at each scan point to provide a coarse and quick overview of the scanned region^3^. Moreover, the resolution of common microscopy techniques can be limited by the beam size, such that features in the sample smaller than the beam size can be hard to detect without performing advanced imaging reconstruction techniques. For weak absorbing objects, obtaining good absorption contrast in hard X-rays is challenging. Since phase gradients in the object can deflect X-rays and result in intensity redistribution on the detector, a few methods based on this mechanism have been proposed to visualize weak absorbing objects. For example, scattering power evaluation was proposed to map out the ratio of scattered photons and incident photons at individual scan positions to give immediate online feedback on the location of a biological specimen^6^; and differential phase contrast obtained by plotting moments of diffraction patterns as a function of positions also provides strong contrast over absorption for objects composed of light elements^7,8^.

Beyond scattering power and moments, more information is embedded in the diffraction patterns. For example, sample feature size, shape, and orientation can be extracted in small-angle X-ray scattering to obtain a deeper understanding of material properties^9,10^. While these techniques provide immediate feedback and mapping of the scattering and transmission properties of the object within the field of view, an automatic and robust method to identify “important” data to be used for the reconstruction is lacking. Therefore, we explore machine learning techniques to achieve automatic data selection without human intervention. In particular, unsupervised learning has drawn considerable attention in the scientific community because of its robustness without requiring labeled data, which is often difficult to acquire because of the lack of ground truth. Unsupervised learning has been successfully applied in different aspects of ptychography, such as identifying probes with diminished quality^11^ or clustering different oxidation behavior inside materials^12^. In this work we utilize the combined knowledge of object transmission and scattering direction information, and we propose a physics-informed unsupervised learning algorithm to identify diffraction patterns at scan positions that are within the RoI. Features that are smaller than the beam size can also be detected and identified through preprocessing of the diffraction patterns by segmenting them azimuthally. As an outcome, only the identified diffraction patterns are used for the reconstruction, saving computational resources while achieving reconstruction quality comparable to that when using the entire dataset.

## Results

Since the ultimate goal is to optimally use the computational resources only on “important” data without sacrificing the reconstruction quality, we now qualitatively examine the reconstruction quality using only the identified diffraction patterns that represent the RoI. Once the RoI is identified (in terms of the scanning positions), we can study the effect of the RoI accuracy on reconstruction quality by adjusting the size of the area (referred to as the “border size”) to be included in reconstruction.

**Figure 1:**
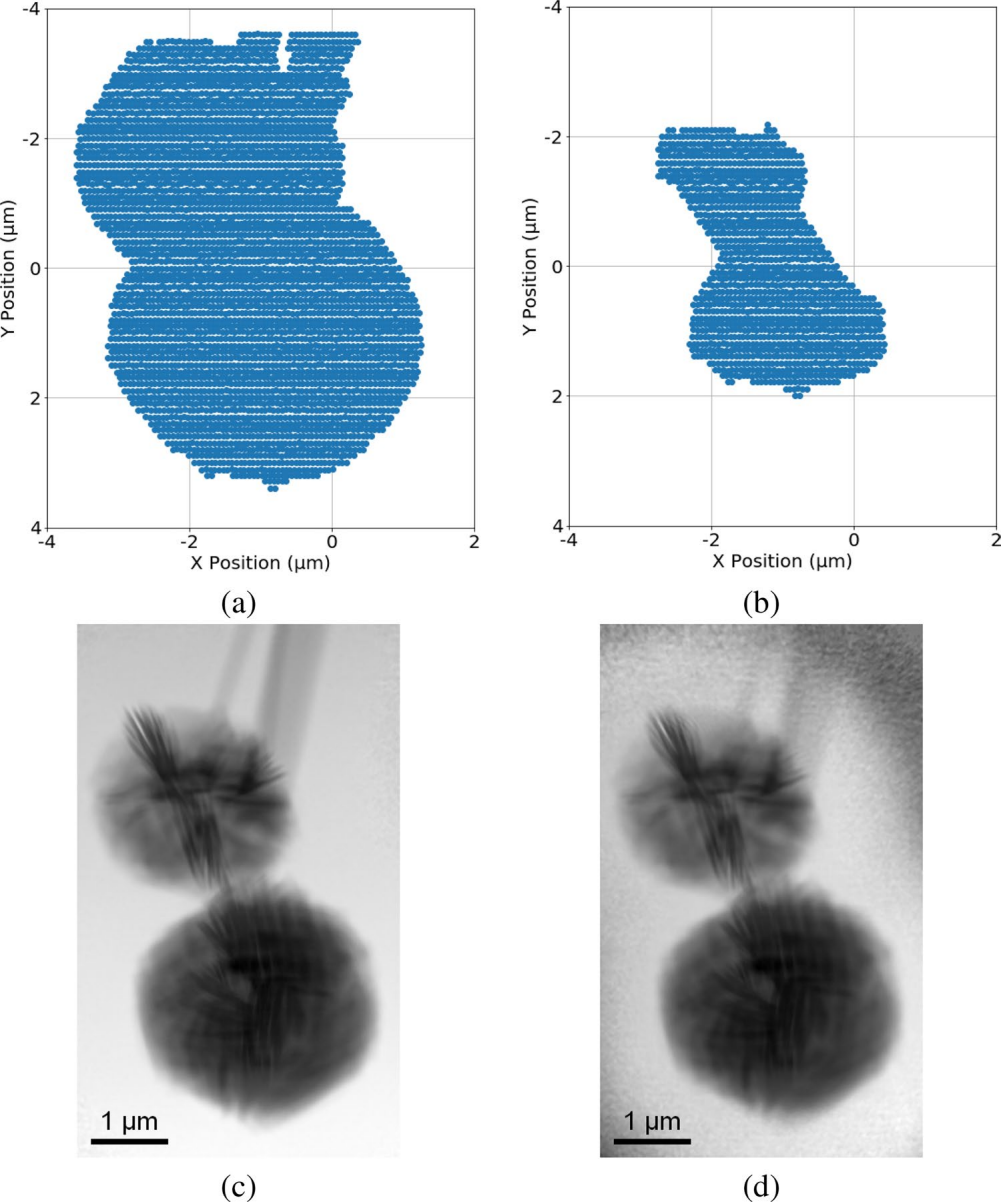
Starting from the identified RoI shown in Fig. 10c: (**a**) & (**c**): expanding the RoI by four step sizes outwards along the xy-directions and its corresponding reconstruction; (**b**) & (**d**): shrinking the RoI by eight step sizes inwards along the xy-directions and its corresponding reconstruction.

As an example, increasing the border size in Fig. 10c by four step sizes outwards along the x-direction and y-direction increases the RoI data from 20% to 26%, as shown in Fig. 1a. Similarly, decreasing the border size by eight step sizes in Fig. 10c inwards along the x-direction and y-direction results in a further reduction of 10% of the total diffraction patterns, as shown in Fig. 1b. The resulting reconstructions by either increasing or decreasing the RoI area are shown in Fig. 1c and d, respectively. We can see that the reconstructions of the true sample area are comparable; however, as expected, the boundary of the FoV shown in Fig. 1d is noisier when compared with Fig. 1c because of the lack of diffraction patterns from these locations.

To quantitatively examine the reconstruction qualities when using only the reduced number of diffraction patterns, we compare the reconstructions between using the full dataset and using our downselected diffraction patterns corresponding to the identified RoI. To be more specific, using the reconstruction image from the full set of diffraction patterns (Fig. 8a) as the reference image, we calculate the structural similarity index measure (SSIM)^13^ between the reference image and other partial reconstructions. We use the state-of-the-art package PtychoShelves^14^ for the reconstruction, while setting the batch size as 10% of the total diffraction patterns, and we perform five independent reconstructions for each test case to obtain an average behavior. All the numerical experiments are implemented in MATLAB and performed on a platform with a single NVIDIA GeForce RTX 2080 GPU.

**Figure 2:**
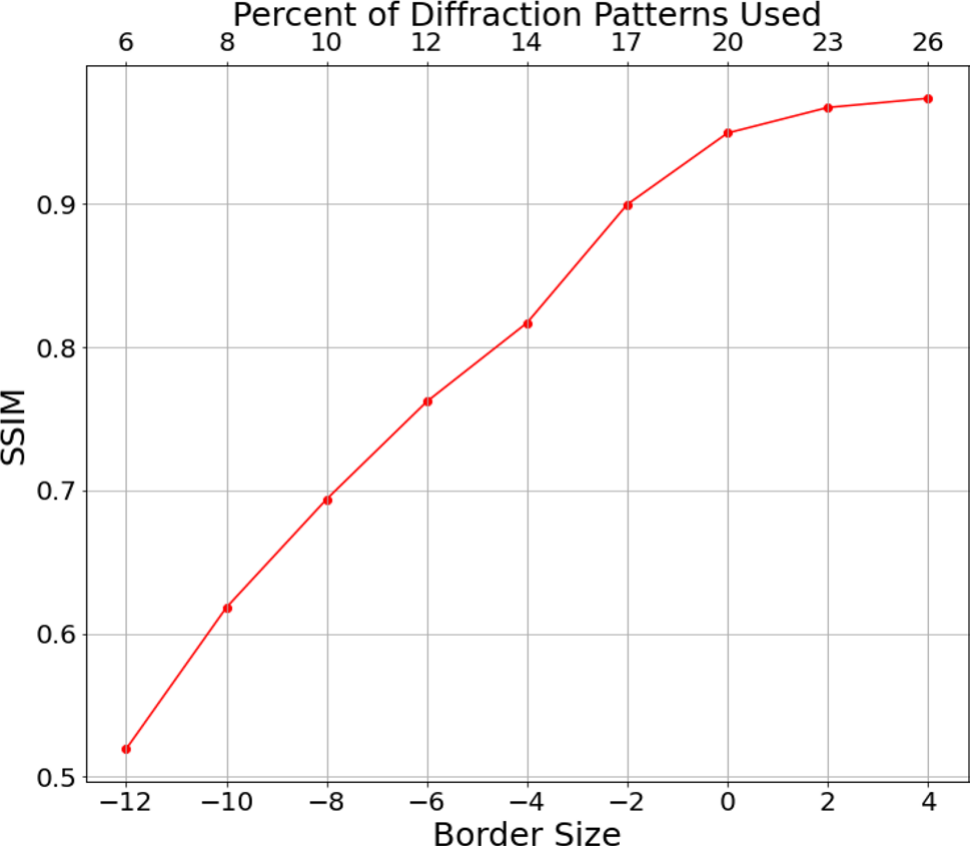
Reconstruction quality measured by SSIM between the reference image (reconstruction using the full dataset) and the reconstruction obtained by different RoIs (as adjusted by different border sizes). Each point is the mean value of five independent reconstructions. Error bars at each data point are not shown due to the minuscule variability and becomes invisible in scale.. A higher border size gives a higher SSIM due to more diffraction patterns surrounding the identified RoI in the reconstruction, whereas a negative border size shrinks the RoI and includes fewer diffraction patterns, therefore yielding a lower SSIM. The identified RoI is robust in the sense that a small adjustment does not change the reconstruction quality significantly.

In Fig. 2, as we decrease the border size to shrink the area of the RoI, the reconstruction quality degrades gradually, as expected. Meanwhile, the SSIM plateaus as we increase the border size to around 2; in other words, increasing the number of diffraction patterns beyond this point yields marginal benefits for the reconstruction quality of the object, thus suggesting the effectiveness of our identified RoI. Next, we provide an empirical complexity analysis to demonstrate the overall computational cost reduction achieved by our proposed method. The time needed for the reconstruction with all diffraction patterns is 104 minutes, whereas running the reconstruction using the retained diffraction patterns (specifically, border size = 0, consisting of around 20% of the total number of the diffraction patterns) as identified with our method takes only 23 minutes, which is roughly only 20% of the time required for the full reconstruction. The total time needed for the preprocessing step to identify the RoI is on the order of 10 seconds, which includes the center of mass (CoM) calculation (10 seconds) and the k-means clustering (less than 1 second). Since almost the entirety of the 10 seconds is spent on calculating the CoM for every diffraction pattern in the dataset, one can conservatively estimate that half a second is needed for every 1000 diffraction patterns in a dataset. Therefore, the preprocessing time in general is negligible when compared with the expensive reconstruction.

### Segmentation-based small feature detection

**Figure 3:**
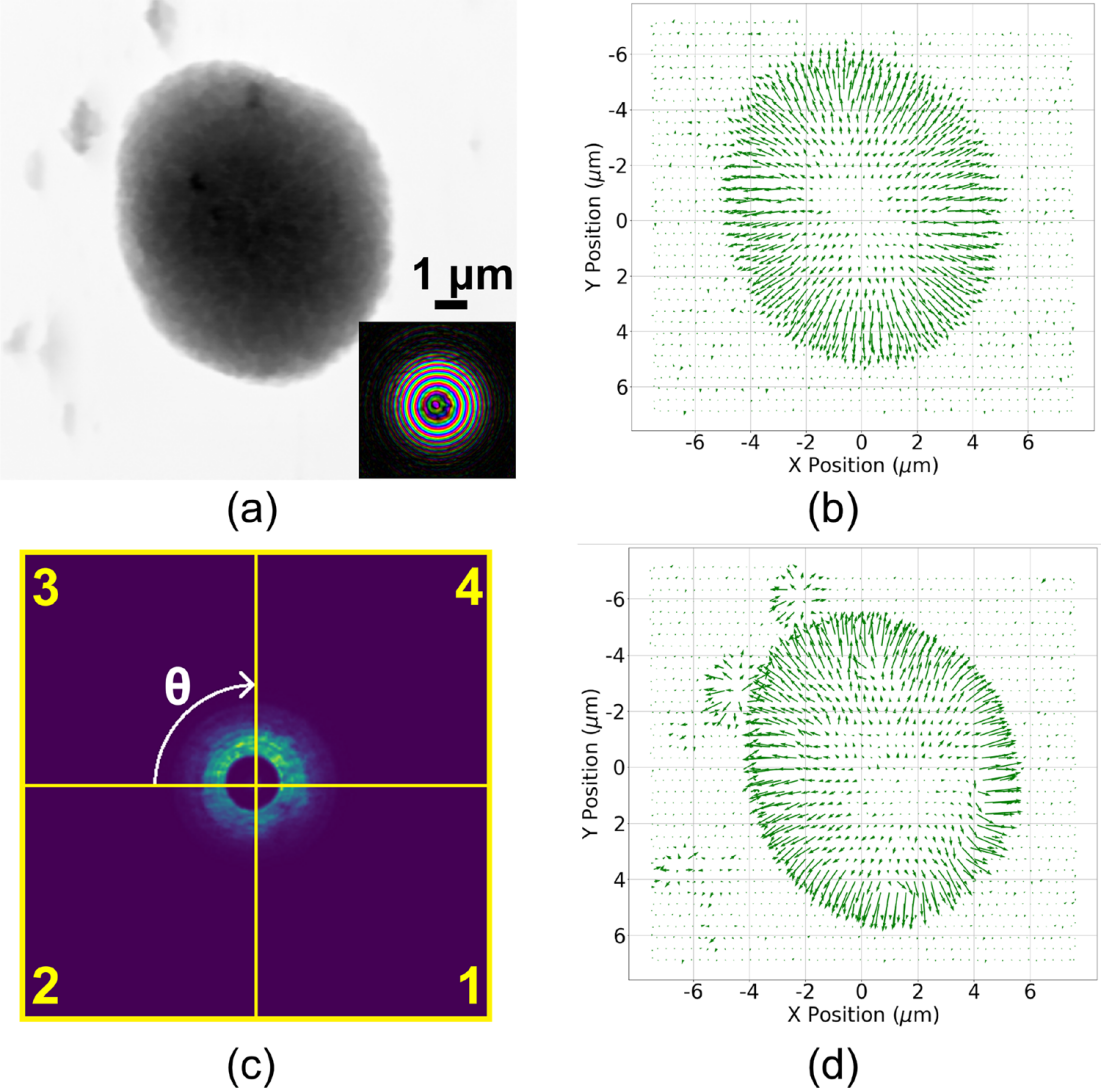
(**a**) Ptychographic reconstruction of a sample with small particles smaller than the beam size: The inset shows the probe size at the same scale. (**b**) Generated CoM scattering direction (quiver) plot using the entire diffraction pattern: Small particles surrounding the main object are not visible. (**c**) Sectioning of the diffraction pattern into four azimuth segments ($\theta = 90^{\circ }$). (**d**) CoM scattering direction plot generated by using only the third segment in (**c**) instead of the entire diffraction pattern: In this case, the small particles are visible when compared with (**b**).

We have demonstrated how combining two different modalities, namely, scattering and absorption, can provide complementary information on sample property; automatically classifying the combined information can further extract the most informative region of interest to accelerate the performance of sample reconstruction. However, calculating the sum or CoM from the entire diffraction pattern to reflect absorption and scattering may fail to represent object features that are smaller than the beam size. Consider the phase reconstruction of the sample shown in Fig. 3a where several small particles surround the main particle situated in the middle. In this sample, the sizes of these surrounding particles are smaller than the beam size, which is shown in the inset. By calculating the CoM in each diffraction pattern and following the same steps as described in the Methods section to generate the CoM quiver plot, which represents the scattering direction at each scan position, shown in Fig. 3b, we see that information about the small particles is lost and not visible in the scattering direction plot and will therefore be undetectable in the following clustering step. In this case, diffraction patterns at scan positions where the small features are present may show anisotropic scattering, due to the interactions of these small features at various orientations. As a result, aggregating this information by considering the diffraction pattern as a whole can average out the calculated CoM, making it difficult to detect a clear scattering direction and, consequently, failing to identify the precise region of interest.

**Figure 4:**
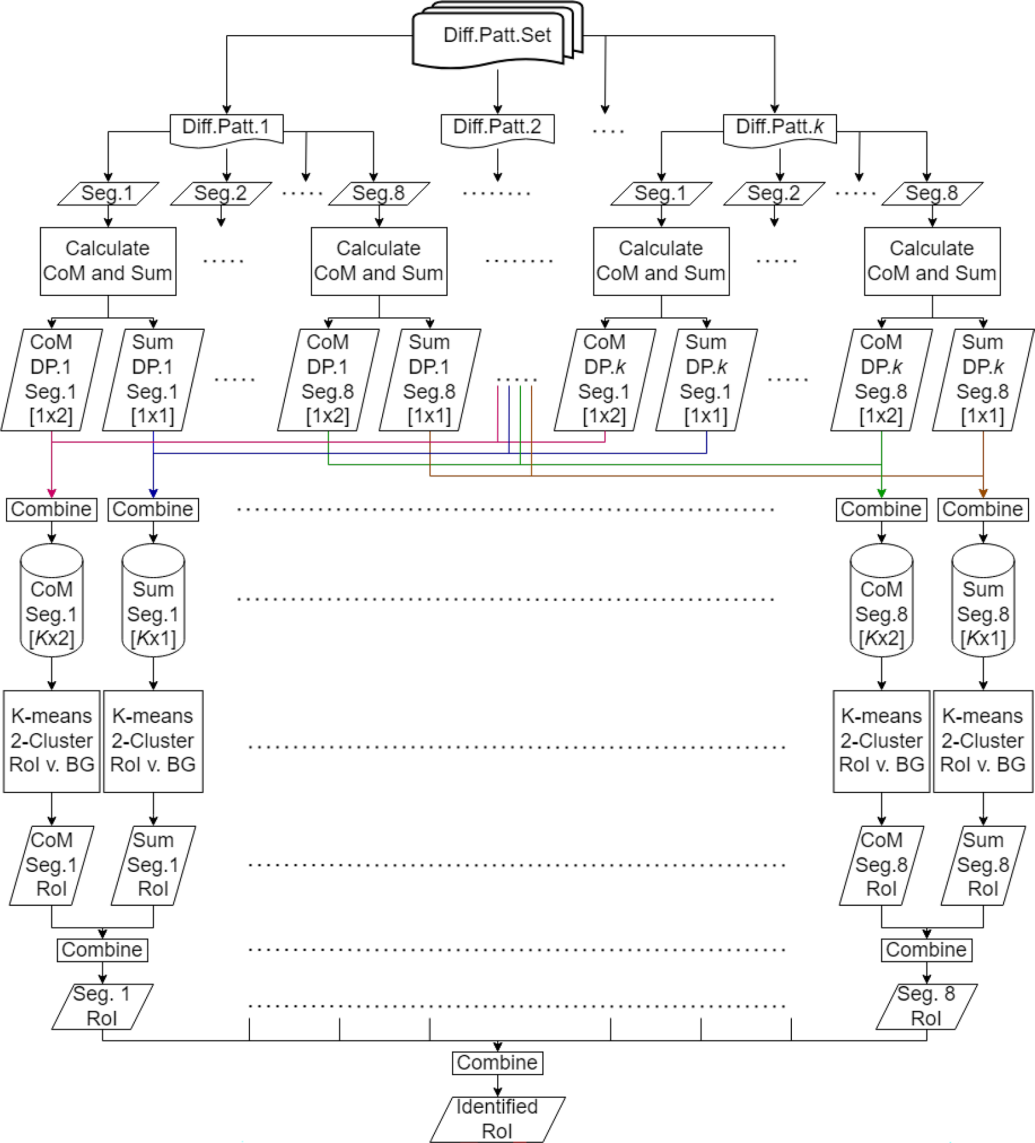
Flowchart outlining the key steps of the proposed method. Using eight segments as an example, each diffraction pattern is first segmented into eight segments, allowing the CoM (i.e., scattering informaiton) and sum (i.e. transmission information) to then be calculated for each segment. We show the dimension of each element in the square brackets. The calculated CoM and sum for each segment from every diffraction pattern are then combined into its respective segment bin, where K-means clustering is then applied to each individual bin to separate RoI and background (BG). The clustered result that identifies the RoI from each bin is ultimately combined to return the final identified RoI.

Drawing inspiration from methods that segment diffraction patterns for differential phase contrast imaging^15,16^, we propose a remedy for this issue by segmenting the diffraction pattern into different azimuthal segments first and then calculating the CoM of each of the segments separately.To clarify, we first divide each diffraction pattern into various azimuthal segments. Next, we calculate the CoM for each of these segments individually. The final scattering plot is obtained by combining the separately calculated scattering plots from each of these individual segments. Key steps for the proposed method have been outlined in the flowchart shown in Fig. 4, which uses eight segments as an example. This method allows the detection of individual scattering directions caused by features that are smaller than beam size within a single diffraction pattern, which can then be leveraged to truthfully determine the final RoI. We provide a quick demonstration by dividing each diffraction pattern into four azimuthal segments, shown in Fig. 3c ($\theta = 90^{\circ }$), and correspondingly calculate each segment’s absorption and CoM instead of the diffraction pattern in its entirety. We can see that, as shown in Fig. 3d, using the third segment of the diffraction pattern as an example, the obtained scattering direction plot can now detect the smaller particles. While we use the third segment as an example, we note that these smaller particles can also be detected when other segments of the diffraction patterns are used.

**Figure 5:**
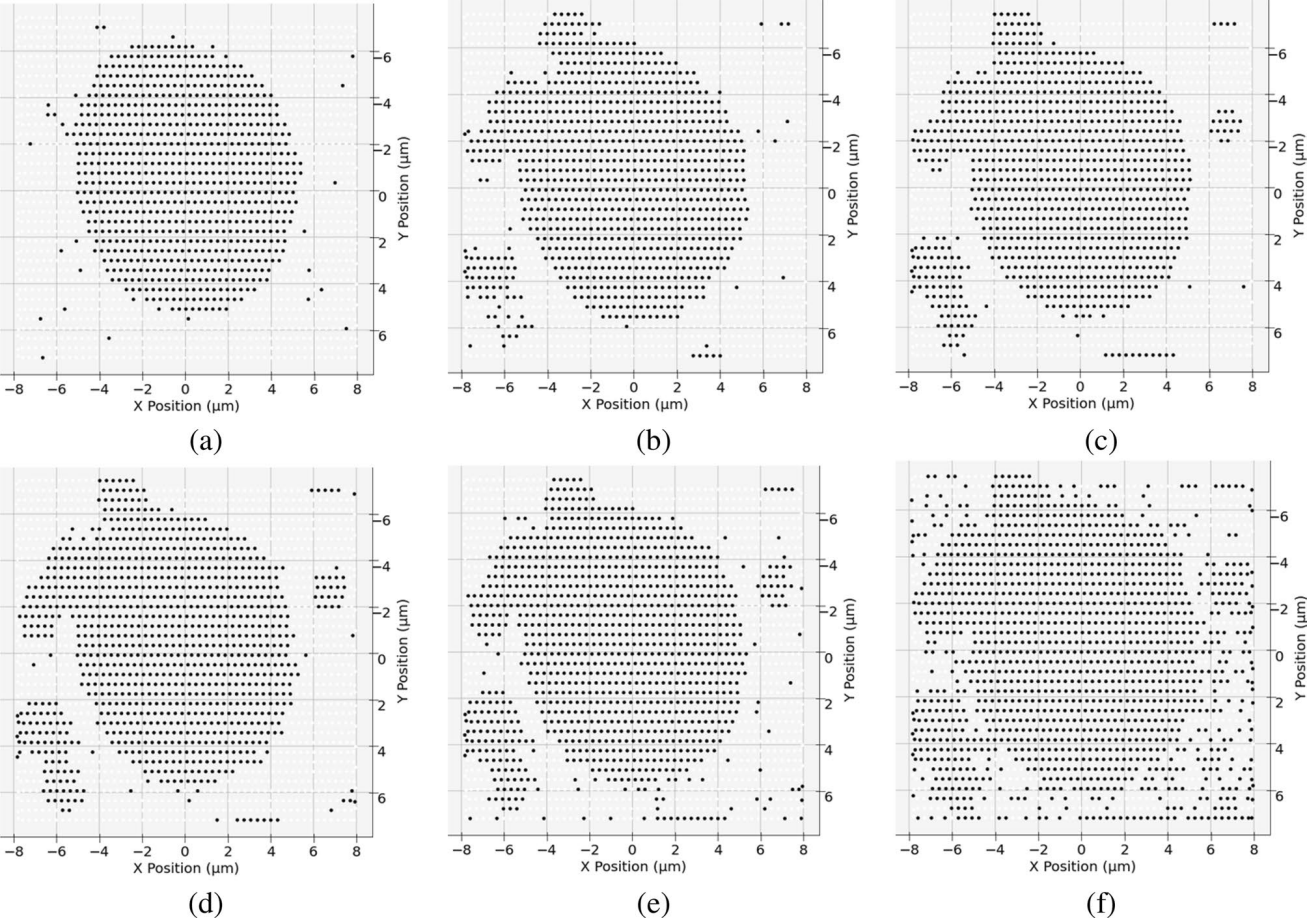
Identified RoI maps as a result of performing clustering on a total of 1,836 preprocessed diffraction patterns (estimated SNR = 6.38), covering an area of 15 × 14 μm (horizontal step size: 0.3 μm, vertical step size: 0.4 μm), which were segmented as follows: (**a**) One segment (i.e., whole diffraction pattern, no segmentation), $42.7\%$ of diffraction patterns identified as within the region of interest. (**b**) Four segments, around $51.2\%$ of diffraction patterns identified as within the region of interest. Particles smaller than beam size to the left of the object are successfully identified. (**c**) Eight segments, $52.2\%$ of diffraction patterns identified as within the region of interest. Another particle smaller than beam size to the right of the object is also successfully identified. (**d**) Sixteen segments, $53.6\%$ of diffraction patterns identified as within the region of interest. Because of the increase in sensitivity each time the number of diffraction pattern segments is increased, more random noise across the field of view is introduced from this point on. (**e**) Twenty-four segments, $57.8\%$ of diffraction patterns identified as within the region of interest. (**f**) Forty segments, $73.9\%$ of diffraction patterns identified as within the region of interest.

Next, we apply the same clustering method as described in the Methods section on each segmented diffraction patterns separately to generate its corresponding RoI map, where each scan point could be labeled as either within the set of RoI points or belonging to the background. Using the example in Fig. 3, in order to incorporate all information from all four segments, we generated four different RoI maps, similar to that shown in Fig. 10c. These RoI maps can then be automatically aligned and combined, where if a scan point is deemed within the RoI using any segment, then it is labeled as such in the final list of scan points within the RoI. For the comparison purposes, RoI clusters are shown for when diffraction patterns are separated into different numbers of segments in Fig. 5. When no segmentation is applied, the resulting clustered RoI (using Fig. 3b) is shown in Fig. 5a, where the small particles are not detected in an easily identifiable manner. Figure 5b shows the final clustered result of when the diffraction pattern is segregated into four segments (seen in Fig. 3c): the small particles to the left are identified and is included into the final list of scan positions within the RoI. As we increase the number of segments to eight ($\theta = 45^{\circ }$), shown in Fig. 5c, the sensitivity increases, and the smaller particle to the right of the object is also successfully detected and identified. However, when the number of segments is increased to sixteen ($\theta = 22.5^{\circ }$) and twenty-four ($\theta = 15^{\circ }$), as seen in Fig. 5d and e, respectively, no new particles show up; instead, noise becomes labeled as RoI as a result of being amplified. Figure 5f shows the result of segregating the diffraction pattern into forty segments ($\theta = 9^{\circ }$). In this extreme case, almost three-quarters of the entire scan positions is labeled as within the RoI, and the information is no longer useful in terms of separating background and object. Segmenting the diffraction pattern into eight segments is sufficient to obtain small particle positions information surrounding the main object while having a clear contrast to the background.

**Figure 6:**
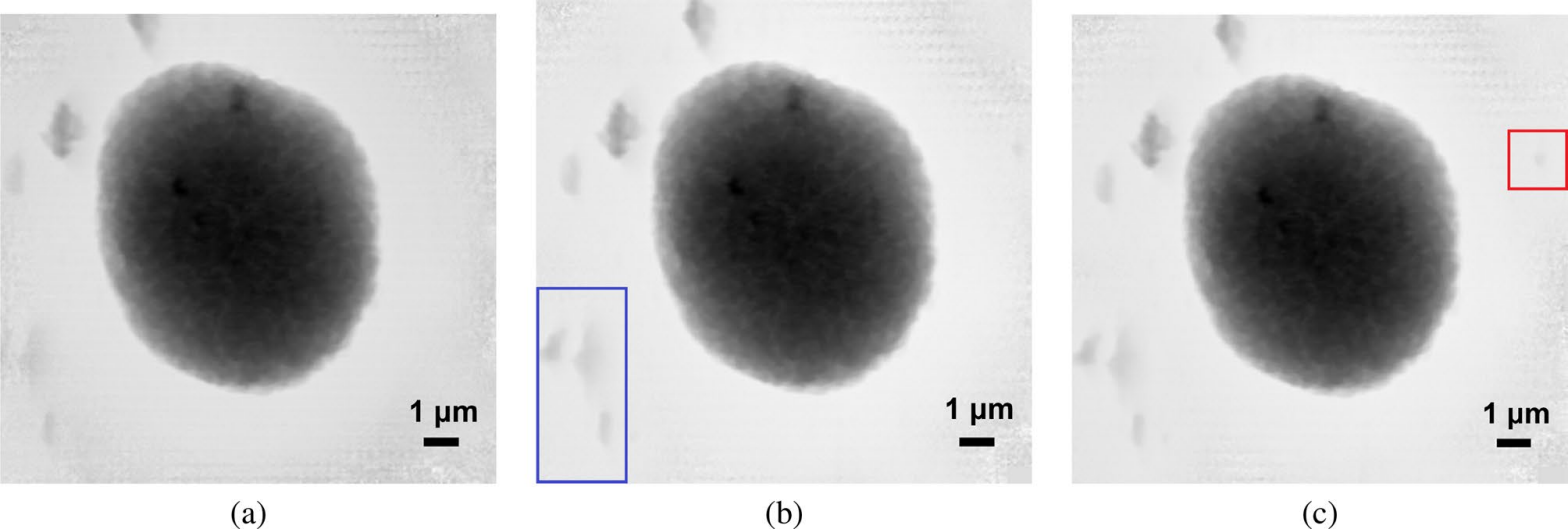
(**a**) Ptychographic reconstruction using diffraction patterns from identified scan positions shown in Fig. 5a, where the clustering algorithm is performed on the whole diffraction pattern. (**b**) Ptychographic reconstruction using diffraction patterns from identified scan positions shown in Fig. 5b, where the clustering algorithm is performed after preprocessing each diffraction pattern by splitting it into four segments. (**c**) Ptychographic reconstruction using diffraction patterns from identified scan positions shown in Fig. 5c, where the clustering algorithm is performed after preprocessing each diffraction pattern by splitting it into eight segments. As the diffraction patterns are gradually split into higher numbers of segments, the reconstruction shows higher contrast between the small particles and the background, as indicated in the colored boxes.

Figure 6 shows the phase reconstruction performed on different numbers of diffraction pattern segmentations (i.e., clustered scan positions shown in Fig. 5a–c). Comparing no segmentation (Fig. 6a), four segments (Fig. 6b), and eight segments (Fig. 6c), we see that as the segments increase, the contrast between the small particles and the background becomes more enhanced, as shown in the blue box in Fig. 6b and the red box in Fig. 6c.

**Figure 7:**
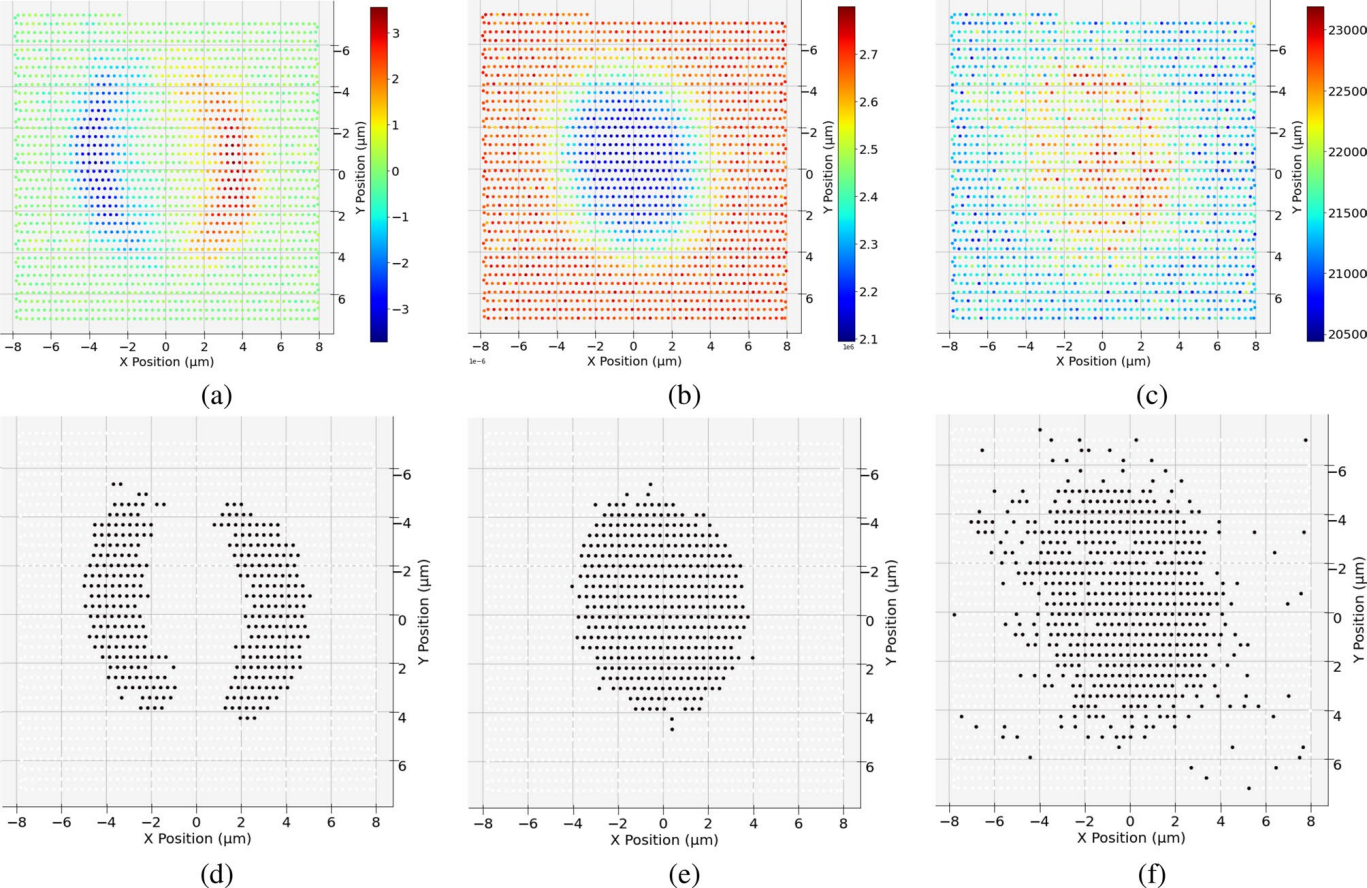
Results from conventional imaging methods: (**a**) Hilbert-differential phase-contrast. (**b**) Scanning transmission X-ray microscopy. (**c**) A computational approach to dark-field imaging, where a circular mask is applied at the center of the diffraction pattern. The pixels outside the mask are summed up and serve as a feature representing scattering sensitivity. (**d**)–(**f**) Estimated RoI using k-means clustering on the data shown in (**a**)–(**c**), respectively. In all three cases, particles surrounding the main object that are smaller than the beam size are not identified when compared with the results of our segmented CoM clustering method shown in Fig. 5c.

We compare our results with imaging results from several conventional methods. The first is Hilbert-differential phase-contrast (HDPC)^17–19^, which shows the differential phase contrast in the horizontal direction, shown in Fig. 7a. The second is scanning transmission X-ray microscopy (STXM)^20,21^, where the absorption contrast is mapped on its corresponding 2D scan position, shown in Fig. 7b. The third is a computational approach toward dark-field imaging^22^, where different methods can be implemented by segmenting the diffraction pattern with circular masks ^23^ or annular masks^7,15^. Here, we divide each diffraction pattern into two regions by applying a circular mask at the center of the diffraction pattern. The circular mask is applied such that the pixels belonging to the direct transmitted beam falls within the mask, and the sum of the values at these pixels can be used as a feature that directly gives absorption sensitivity. The result is identical to STXM. Similarly, the pixels that fall outside the mask belong to the high-frequency region, and the summation of the values at these pixels is attributed to the scattering from the object and directly gives the scattering sensitivity, as shown in Fig. 7c. To provide a comparison for estimating the RoI for these methods with our proposed segmented CoM clustering method, we apply the same k-means clustering algorithm on each of these images obtained by the above methods. After taking the absolute values of the data shown in Fig. 7a, we apply k-means clustering to separate this dataset into two clusters: the background and the RoI of the object. The results are shown in Fig. 7d. Figure 7e was obtained by applying k-means on the absorption sensitivity data shown in Fig. 7b into two clusters, and Figure 7f was obtained the same way by applying k-means directly on the data shown in Fig. 7c. In all three cases, particles smaller than the beam size are not identified when compared with the results of our segmented CoM clustering method as shown in Fig. 5c.

## Discussion and conclusion

We proposed an unsupervised learning framework to successfully classify the “important” data corresponding to the region of interest, which ultimately leads to a decrease in time for the reconstruction while preserving high reconstruction quality. More importantly, we exploit information provided by different data modalities that are correlated but also complementary. For example, the absorption contrast is mostly effective for identifying regions with high optical density but fails in regions with low optical density. On the other hand, the scattering orientation information, in the form of CoM of each diffraction pattern, excels in detecting scattered light changes, which is effective in regions with feature changes. The combined use of multimodal information from both the absorption contrast and scattering orientation, as directly extracted from diffraction patterns, complements the otherwise incomplete object information. In cases when a object is weakly absorbing, and the absorption contrast may fail in providing the needed complementary data, the scattering data will still succeed in providing boundary information. Depending on the need, an additional “fill-in” function can be included in the algorithm to fill-in areas within the identified boundary based on scattering information, and include them towards the RoI for reconstruction. To perform classification, we demonstrate that k-means provides stable results for different samples, and two clusters are chosen to efficiently separate object from background. Although two clusters is sufficient towards identifying object and empty background, even if the object consists of different materials, a worthwhile direction is to enhance our method beyond identifying the current two clusters. For example, by increasing the number of partition clusters, different materials can be successfully identified due to the difference in their corresponding absorption and scattering properties. However, a robust method to estimate the optimal number of clusters needed that best represents different materials within the field of view will require further investigation. While selecting a threshold for the calculated features can be considered trivial in order to determine or adjust the RoI instead of using K-means, but would still require some form of human intervention as opposed to our proposed method. Our presented unsupervised classification method can be valuable in situations when, for example, when hundreds or thousands of scans of the same sample are taken over a period of time to investigate the changes of the sample over time. In these cases, manually selecting different thresholds and variables for each scan can become an issue if human judgement is needed. We further demonstrate the application of our proposed method on experimental datasets and show that with the identified “important” diffraction patterns, the total processing time including any preprocessing and reconstruction can be dramatically reduced without sacrificing reconstruction quality. More importantly, we demonstrate that by segmenting the diffraction patterns, smaller particles compared with the beam size can also be detected and identified. Our developed method has the potential of solving problems that may benefit from obtaining an immediate preview of the resulting reconstruction, detecting features that are smaller than the beam size, identifying diffraction patterns that relate to the region of interest, and removing unwanted data prior to committing time toward reconstruction. As one future direction, instead of performing a simple raster scan over the entire field of view, we can extend our proposed method to provide feedback on “important” data in real time and facilitate the optimal experimental scanning pattern based on the morphology of the object, in order to save the overall data acquisition time. Our presented method is currently demonstrated on isolated-type samples that are commonly required for tomography, as for 2D ptychography or ptychographic laminiography^24^ of extended samples which don’t have featureless backgrounds, different contrast mechanisms (such as simultaneously acquired correlated fluorescence signals) to better extract the ROIs will be explored and built into the list of classification methods.

## Methods

We describe *K* diffraction patterns $\textbf{P}^{k} \in \mathbb {R}^{N\times M}$, $(k=1,\cdots ,K)$, where $N\times M$ is the corresponding diffraction pattern size. The two-dimensional CoM array can then be calculated as

1 $$\begin{aligned} \left( O_{x}^k,O_{y}^k\right) = \left( \frac{1}{T^{k}}\sum _{i=1}^{N}i\sum _j \textbf{P}_{j,i}^k, \, \frac{1}{T^{k}}\sum _{j=1}^{M}j\sum _i \textbf{P}_{j,i}^k\right) , \end{aligned}$$

where $T^{k}$ is the sum of all pixel values over the entire diffraction pattern, namely, $T^k=\sum _{i,j}\textbf{P}_{i,j}^k$. Calculating the CoM according to Eq. (1) for *K* diffraction patterns returns $\textbf{O} \in \mathbb {R}^{K\times 2}$, whose row *k* represents the (*x*, *y*) coordinates of the CoM of the *k*th diffraction pattern.

To calibrate for beam-center offset errors and to represent each row of $\textbf{O}$ as a vector having an origin in the calibrated center of the image, we transform $\textbf{O}$ to its standard normal distribution form $\vec {\textbf{O}}$ with zero mean and unit variance as

2 $$\begin{aligned} \vec {\textbf{O}}=\left[ \left( \vec {O}_{x}^k,\vec {O}_{y}^{k}\right) \right] _{k=1}^K = \left[ \left( \frac{O_{x}^k-\bar{O}_{x}}{\sigma _{x}},\frac{O_{y}^k-\bar{O}_{y}}{\sigma _{y}}\right) \right] _{k=1}^K, \end{aligned}$$

where $\left( \bar{O}_{x},\bar{O}_{y}\right)$ and $\left( \sigma _{x},\sigma _y\right)$ are the mean and standard deviation of the first and second columns of $\textbf{O}$, respectively. The magnitude of each row vector of $\vec {\textbf{O}}$ is correlated with the strength of the scattered light caused by the object at scanning position *k* and yields characteristic information regarding the outline and shape of the object.

**Figure 8:**
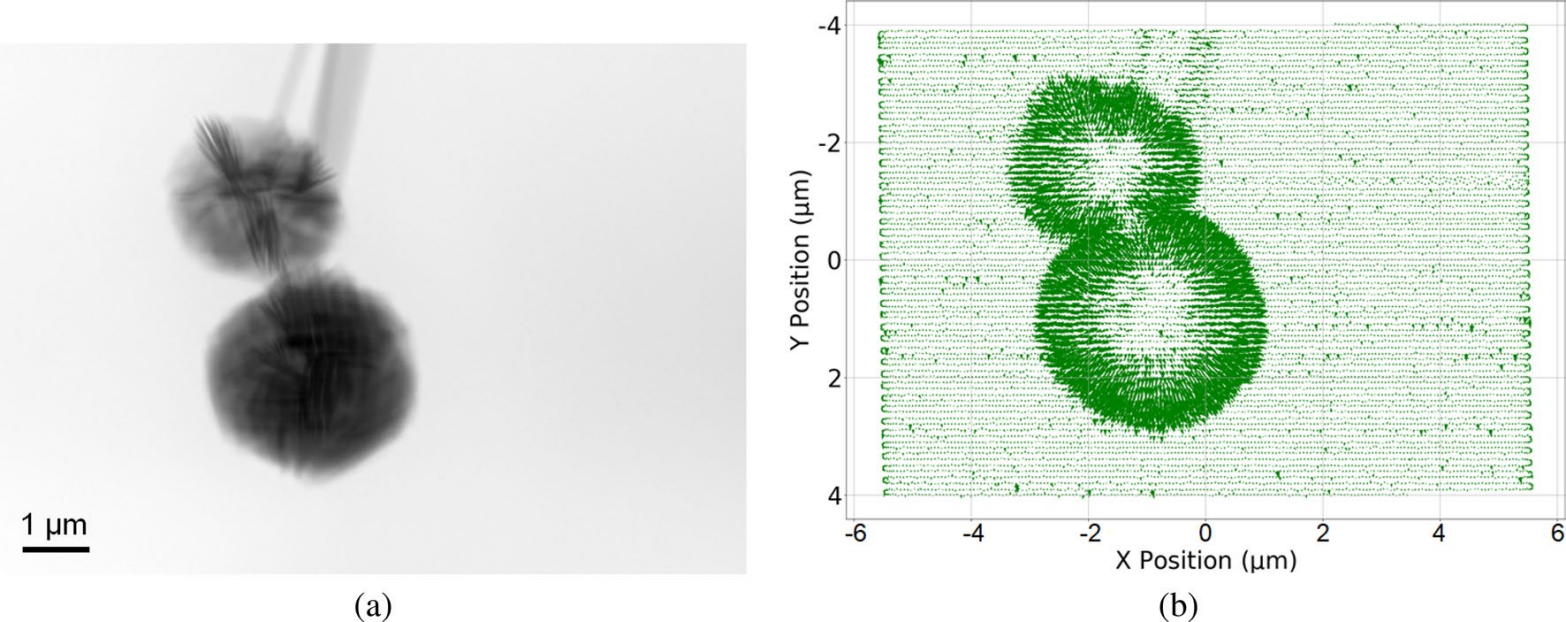
(**a**) Reconstruction with all diffraction patterns. (**b**) Quiver plot representing the calculated CoM vector for each diffraction pattern at its scan position. This serves as a map for the scattering direction information of the object and highlights the feature change area, including the multiple bands at the top of the image.

**Figure 9:**
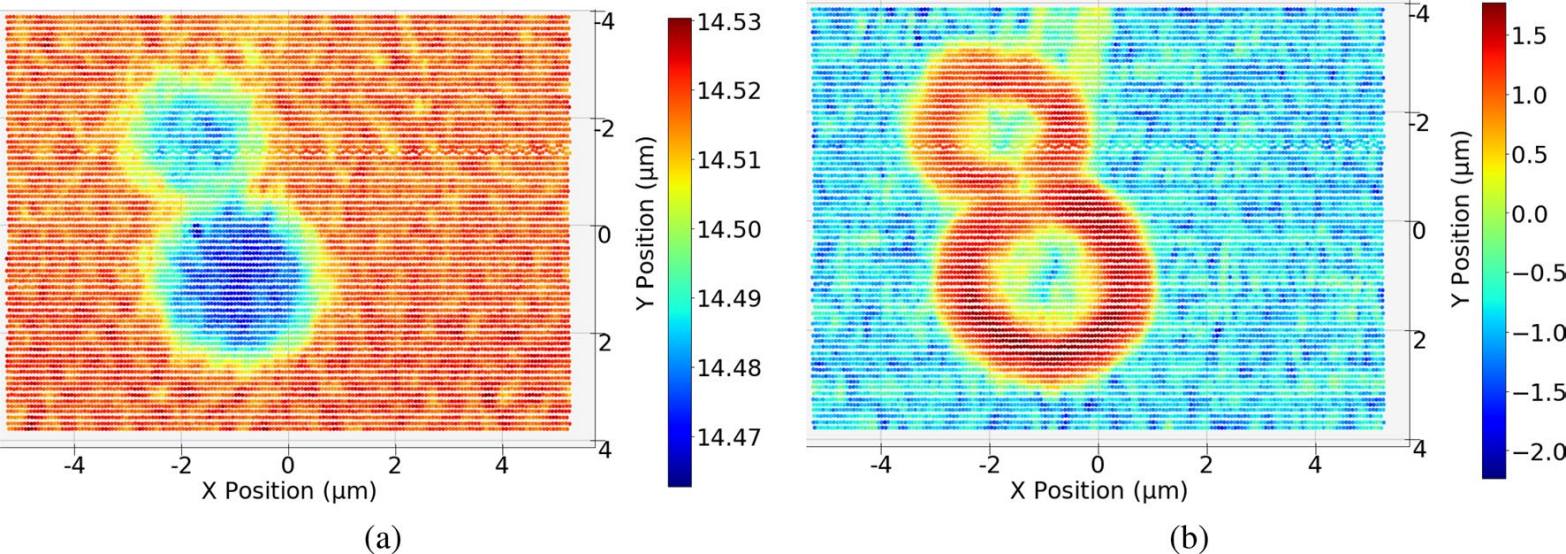
(**a**) Scatter plot of the natural log value of the sum of all pixels in each diffraction pattern at its scanning position. (**b**) Scatter plot of the natural log value of the magnitude of each arrow in the scattering direction plot seen in Fig. 8b.

We demonstrate the characteristic information embedded in the CoM array of an experimental dataset that was acquired by the Velociprobe instrument^25^ at the Advanced Photon Source. CuS secondary particles composed of a batch of nanosheets were imaged at 8.8 keV with the fly-scan mode (probe size: $\sim$1 μm in diameter), in which the object is moved continuously as a series of diffraction patterns acquired by an Eiger X 500K detector. A total of $K = 15,980$ diffraction patterns, covering an area of 11 × 8 μm (horizontal step size: 0.06 $\mu m$, vertical step size: 0.1 $\mu m$), at an estimated SNR of 5.6, were collected at a continuous frame rate of 200 Hz in less than 80 seconds. Each diffraction pattern was cropped to 256 $\times$ 256 and then all the cropped diffraction patters were used for reconstruction by the generalized least-squares maximum likelihood algorithm^26^ implemented in the PtychoShelves package^14^. Figure 8a shows the reconstructed phase using the complete dataset. The CoM vector array for this dataset is calculated by using Eq. (2) and is shown in Fig. 8b as a quiver plot, providing a map for the scattering direction of the object. As a complementary modality to the oriented scattering information present in the CoM array, we further exploit the absorption contrast image, as obtained from the total signal recorded on the pixelated area detector at each scan position, $T^k$. In order to filter out noisy data, a mean filter of size 3 × 3 is applied to both the absorption contrast image and the CoM magnitude image, which are shown in base-*e* log scale in Fig. 9a and b, respectively.

**Figure 10:**
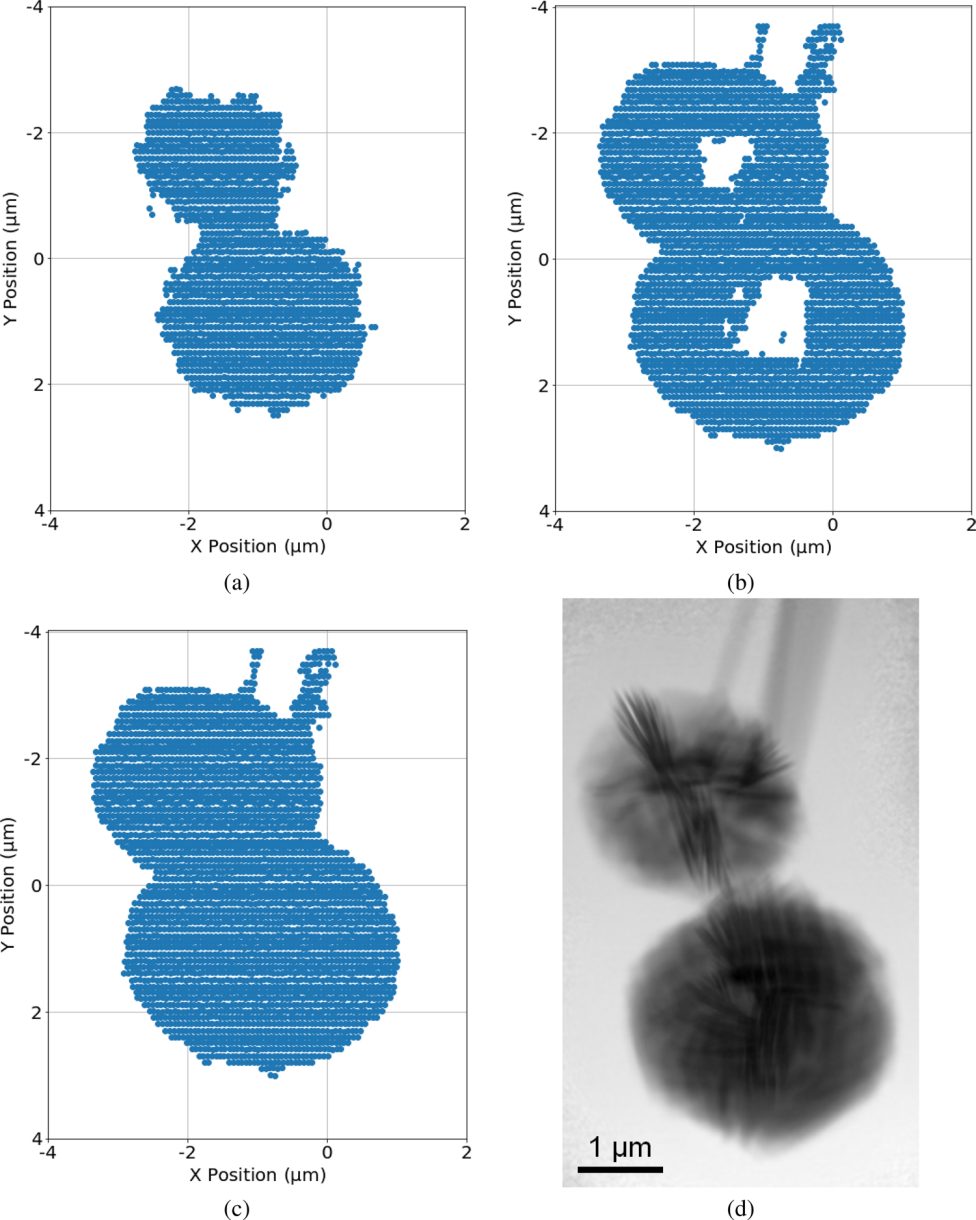
(**a**) RoI cluster identified as high absorption region. (**b**) RoI cluster identified as high scattering region. (**c**) Combined RoI clusters (**a**) and (**b**). (**d**) Reconstruction of object using only retained diffraction patterns in (**c**).

Given the characteristic information embedded in these two complementary modalities, we now explore an unsupervised clustering technique to classify different types of features. First, we apply k-means^27^ (10 iterations) to partition the absorption contrast into two clusters. The cluster with lower values corresponds to diffraction patterns with lower light transmission due to the object absorption, and vice versa. A total of 1832 diffraction patterns are identified and labeled as blue dots in their corresponding scan position in Fig. 10a, which constitutes the first part of the RoI cluster. Similarly, we apply k-means to partition the magnitudes of the CoM array into two clusters. The cluster of diffraction patterns with higher CoM magnitude corresponds to higher gradient changes in terms of scattering strength, which reflects the outline of the RoI as the second part of the RoI cluster. In this dataset, a total of 2998 diffraction patterns belonging to this cluster are identified and shown in Fig. 10b. In the final step, we combine these two identified clusters to obtain the complete RoI cluster; counting overlapping diffraction patterns only once, a total of 3260 diffraction patterns are obtained, reducing the RoI data to 20% of the total data, as shown in Fig. 10c. The corresponding reconstruction using only the identified diffraction patterns from the RoI is shown in Fig. 10d.

## Data Availability

Data underlying the results presented in this paper are not publicly available at this time but may be obtained from the corresponding author upon reasonable request.
